# The Modulatory Properties of Chronic Antidepressant Drugs Treatment on the Brain Chemokine – Chemokine Receptor Network: A Molecular Study in an Animal Model of Depression

**DOI:** 10.3389/fphar.2017.00779

**Published:** 2017-11-01

**Authors:** Ewa Trojan, Joanna Ślusarczyk, Katarzyna Chamera, Katarzyna Kotarska, Katarzyna Głombik, Marta Kubera, Agnieszka Basta-Kaim

**Affiliations:** Department of Experimental Neuroendocrinology, Institute of Pharmacology, Polish Academy of Sciences, Kraków, Poland

**Keywords:** CXCL12, CX3CL1, chemokine receptors, hippocampus, frontal cortex, prenatal stress, antidepressant drugs, TGFβ/Smad pathway

## Abstract

An increasing number of studies indicate that the chemokine system may be the third major communication system of the brain. Therefore, the role of the chemokine system in the development of brain disorders, including depression, has been recently proposed. However, little is known about the impact of the administration of various antidepressant drugs on the brain chemokine – chemokine receptor axis. In the present study, we used an animal model of depression based on the prenatal stress procedure. We determined whether chronic treatment with tianeptine, venlafaxine, or fluoxetine influenced the evoked by prenatal stress procedure changes in the mRNA and protein levels of the homeostatic chemokines, CXCL12 (SDF-1α), CX3CL1 (fractalkine) and their receptors, in the hippocampus and frontal cortex. Moreover, the impact of mentioned antidepressants on the TGF-β, a molecular pathway related to fractalkine receptor (CX3CR1), was explored. We found that prenatal stress caused anxiety and depressive-like disturbances in adult offspring rats, which were normalized by chronic antidepressant treatment. Furthermore, we showed the stress-evoked CXCL12 upregulation while CXCR4 downregulation in hippocampus and frontal cortex. CXCR7 expression was enhanced in frontal cortex but not hippocampus. Furthermore, the levels of CX3CL1 and CX3CR1 were diminished by prenatal stress in the both examined brain areas. The mentioned changes were normalized with various potency by chronic administration of tested antidepressants. All drugs in hippocampus, while tianeptine and venlafaxine in frontal cortex normalized the CXCL12 level in prenatally stressed offspring. Moreover, in hippocampus only fluoxetine enhanced CXCR4 level, while fluoxetine and tianeptine diminished CXCR7 level in frontal cortex. Additionally, the diminished by prenatal stress levels of CX3CL1 and CX3CR1 in the both examined brain areas were normalized by chronic tianeptine and partially fluoxetine administration. Tianeptine modulate also brain TGF-β signaling in the prenatal stress-induced animal model of depression. Our results provide new evidence that not only prenatal stress-induced behavioral disturbances but also changes of CXCL12 and their receptor and at less extend in CX3CL1–CX3CR1 expression may be normalized by chronic antidepressant drug treatment. In particular, the effect on the CXCL12 and their CXCR4 and CXCR7 receptors requires additional studies to elucidate the possible biological consequences.

## Introduction

Depression is a common and serious mental disorder that affects nearly 350 million people worldwide (Murray et al., 2013). Despite the significant social burden that stems from this disease, there are still significant gaps in our scientific understanding of the biological basis and progression of this illness. Currently, psychiatric disorders, including depression, are believed to have a multifactorial origin that involves molecular, cellular, structural and functional dysfunctions in various brain areas, which makes the task of understanding the background of depression based on one hypothesis impracticable (Krishnan and Nestler, 2008). Due to the complexity of depression, unsurprisingly, pharmacotherapy directed toward one particular mechanism is often marginally effective. This issue explains why current antidepressant drug treatment is usually effective in only 50% of patients, and clinical data show that patients respond to this medication only after weeks or months of chronic treatment (Masi and Brovedani, 2011). Moreover, there is major socio-economic pressure to find new, attractive targets for developing more effective strategies (Papakostas and Ionescu, 2015).

A growing body of evidence indicated that beyond the reciprocal influence of genetic and environmental factors, depressive symptoms are frequently associated with inflammatory processes (Dowlati et al., 2010; Kubera et al., 2011; Maes, 2011; Liu et al., 2012; Ogłodek et al., 2014). Among immune mediators, chemokines (chemotactic cytokines) are the main proteins responsible for the regulation of inflammatory processes in the brain. Chemokines are a group of small (8–14 kDa) polypeptides, which mediate their biological effects *via* interactions with 7-transmembrane G protein-coupled receptors. Their basic role in the periphery is the recruitment of leukocytes to maintain the function of the immune system (Gerard and Rollins, 2001; Moser et al., 2004). However, in the brain, data indicate that these proteins are also involved in the modulation of nervous system functions and in the restoration and maintenance of brain homeostasis (Stuart et al., 2015). Through the activation of diverse signaling pathways, chemokines regulate migration (Yin et al., 2013; Bardina et al., 2015; Merino et al., 2015), proliferation of neuronal stem/progenitor cells (Peng et al., 2007; Patel et al., 2012), control axon elongation (Pujol et al., 2005), synaptic pruning processes (Paolicelli et al., 2011) and blood–brain barrier (BBB) permeability. In addition to their role in neuromodulation, studies have shown the significance of chemokines in regulating neuroendocrine functions and mediating the activity of specific neurotransmitter and neuropeptide systems (Heinisch and Kirby, 2009; Di Castro et al., 2016). Recently, researchers have found that some chemokines are key in maintaining the interaction between neuronal and glial cells in both the developing and adult brain (Gómez-Gonzalo et al., 2017; Zhang et al., 2017). When the diverse activity of chemokines in brain was taken into account, chemokines were divided into categories based on their biological activity.

CX3CL1 (fractalkine) and CXCL12/SDF-1α (stromal cell-derived factor-1α) are among the homeostatic chemokines that are constitutively expressed in the brain. CX3CL1 shows higher expression in the brain than in the periphery. Complementary expression of CX3CL1 mainly on neurons and CX3CR1 on microglia establishes a unique communication system between these cells, where CX3CL1–CX3CR1 signaling is responsible for control of microglial activation (Lyons et al., 2009). Importantly, our previous data demonstrated the anti-inflammatory properties of CX3CL1 as well as its important role in the regulation of behavioral disturbances in an animal model of depression (Ślusarczyk et al., 2016). Another constitutively expressed chemokine in the brain is CXCL12 – a CXC-chemokine, which modulates the immune response, but its impact on neuronal development and plasticity and involvement in anxiety disorders mediation should be taken into account (Yang et al., 2016). In addition to the classical CXCR4 receptor, CXCL12 acts also *via* atypical CXCR7, which may operate as a β-arrestin-biased receptor.

Considering the chemokine system as the third major communication system of the brain, this study examine changes in expression of chemokine and chemokine receptors in animal model of depression. Moreover, we evaluated the impact of chronic administration of various antidepressant drugs: tianeptine – an atypical antidepressant, which enhances re-uptake of serotonin; venlafaxine – a serotonin – norepinephrine reuptake inhibitor (SNRI); and fluoxetine – a selective serotonin reuptake inhibitor (SSRI), on the gene expression and protein levels of CX3CL1 and CXCL12 and their receptors CX3CR1, CXCR4, CXCR7 in the hippocampus and the frontal cortex of adult rats in an animal model of depression. Taking into account previous data (Chen et al., 2002) that indicated a role of transforming growth factor β (TGF-β) signaling in the modulation of some chemokine receptor levels, particularly CX3CR1, and in search of the underlying mechanism of the impact of antidepressant drug treatment on chemokine receptor dysfunction in depression, we also focused on the activation of the canonical intracellular pathways linked to TGFβ and its receptors TGFβr1 and TGFβr2, such as phosphorylated Smad2/3, as well as Smad4 and Smad7 levels in the hippocampus and the frontal cortex of adult rats in an animal model of depression.

In the present study, we used a universally recognized animal model of depression based on a prenatal stress procedure (Morley-Fletcher et al., 2003). In this model, behavioral changes and abnormalities in the function of the neuroendocrine system were observed, which were normalized by chronic antidepressant treatment (Szymańska et al., 2009; Budziszewska et al., 2010). Our previous study found that prenatal stress impairs the activity of the immune system not only in the periphery but also in the hippocampus and the frontal cortex, which are important brain areas in the pathogenesis of depression, leading to prolonged microglial activation and malfunction in the chemokine-chemokine receptor axis in adult offspring (Szczesny et al., 2014; Ślusarczyk et al., 2016).

## Materials and Methods

### Animals

Sprague-Dawley rats (200–250 g upon arrival) that were obtained from Charles River (Sulzfeld, Germany) were maintained under standard conditions (at room temperature of 23°C, 12/12 h light/dark cycle, lights on at 06:00 am), with food and water available *ad libitum*. Two weeks after arrival, vaginal smears were obtained daily from the female rats to determine the phase of the oestrous cycle. On the pro-oestrus day, the females were placed with males for 12 h and subsequently checked for the presence of sperm in vaginal smears. Pregnant females were randomly assigned to control and stress groups (*n* = 10 in each group). All experimental protocols were approved by the Committee for Laboratory Animal Welfare and Ethics of the Institute of Pharmacology, Polish Academy of Sciences, Cracow and met the criteria of the International Council for Laboratory Animals and Guide for the Care and Use of Laboratory Animals Consent procedure: 1037/2013.

### Stress Procedure

**FIGURE 1:**
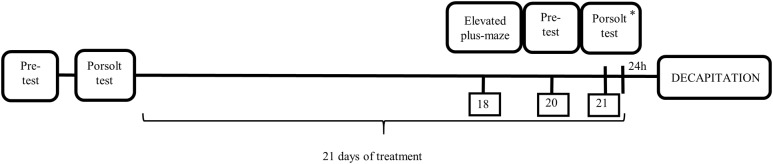
Schematic diagram representing the schedule of the experiment. ^∗^ After Porsolt Test animals were treated with the last dose of antidepressants.

The prenatal stress procedure was performed as previously described (Morley-Fletcher et al., 2003). Briefly, pregnant females were subjected to three stress sessions daily, beginning on the 14th day of pregnancy and continuing until delivery. At 9:00 am, 12:00 pm and 5:00 pm, the rats were placed in plastic cylinders (7 cm × 12 cm) and exposed to bright light (150 W, 1800–2000 lx). Control, pregnant females were left undisturbed in their home cages. Male offspring were selected from 21-day-old litters for the experiment. They were housed in groups of five animals per cage (one or two animals from each litter) under standard conditions. At 3 months of age, the offspring of the control and stressed mothers underwent the first behavioral verification in the forced swim test (**Figure 1**).

### Forced Swim Test (FST)

The forced swim test (FST, Porsolt test) was conducted according to a previously described method (Detke et al., 1995). Briefly, the animals were individually subjected to two trials during which they were forced to swim in a cylinder (50 cm high, 18 cm in diameter) filled with water (23°C) to a height of 35 cm. There was a 24-h interval between the first (pre-test) and second (test) trial. The first trial lasted 15 min, and the second trial lasted 5 min. The total durations of immobility, mobility (swimming) and climbing were measured throughout the second trial (Porsolt et al., 1978; Detke et al., 1995).

### Antidepressant Drug Administration

After the behavioral verification, the control and prenatally stressed offspring were divided into eight experimental groups (CONTROL+VEH, CONTROL+FLU, CONTROL+VEN, CONTROL+TIA, STRESS+VEH, STRESS+FLU, STRESS+VEN, STRESS+TIA; six animals per group) and were treated with antidepressant drugs for 21 days. Fluoxetine (Eli Lilly, Indianapolis, IN, United States), venlafaxine (Sequoia Research, United Kingdom), and tianeptine (Tocris Bioscience, United Kingdom) were intraperitoneally injected once per day between 9:00 am and 10:00 am at a dose of 10 mg/kg, which was diluted in 0.9% saline. The CONTROL+VEH and STRESS+VEH groups received 0.9% saline (Polpharma, Poland). For pharmacological verification of the animal model of depression, animals underwent the elevated plus-maze and again forced swim procedures on the last days of chronic antidepressant drug treatment (according to the schedule illustrated in **Figure 1**).

### Elevated Plus-Maze Test

The elevated plus-maze test was performed as previously described (Pellow et al., 1985). The maze was elevated to a height of 50 cm above the floor and illuminated from below by a dim light (15 W). To allow the animals to adapt to the experimental conditions, they were placed in the experimental room for 1 h before the test. Each subject was individually placed in the central area of the maze facing the closed arm and observed for 5 min. The results are presented as the average number of entries into the open arms and the time in seconds (s) spent in the open arms. An entry was recorded when the animal entered the arm with all four limbs. The behavioral study was not blinded.

### Tissue Collection

Twenty-four hours after the last injection of antidepressant drugs, the animals were sacrificed under non-stress conditions by rapid decapitation. The frontal cortices (FCx) and hippocampi (Hp) were dissected onto ice-cold glass plates, and the tissues were frozen on dry ice and stored at -80°C (for ELISA and Western blot assays) or at -20°C in RNALater^®^ solution (Applied Biosystems, United States) prior to total RNA extraction.

### Tissue Preparation and Determination of Protein Concentration

All tissue samples were placed in 2-ml Eppendorf^®^ tubes filled with an appropriate buffer and homogenized using a Tissue Lyser II (Qiagen, Inc., Valencia, CA, United States). The samples were aliquoted and stored at -20 to -80°C until use to avoid freeze–thaw cycles. In all experiments, the protein concentrations of the analyzed samples were determined using a BCA Protein Assay Kit (Sigma–Aldrich, St. Louis, MO, United States). Protein concentrations were measured at a wavelength of 562 nm using a Tecan Infinite 200 Pro spectrophotometer (Tecan, Mannedorf, Germany) in triplicates for each sample using bovine serum albumin as a standard.

### Quantitative Real-Time Polymerase Chain Reaction (qRT-PCR)

Total RNA was isolated using a RNeasy Mini Kit (Qiagen, Hilden, Germany). The samples were homogenized in an appropriate volume of the lysis buffer supplied with the kit, and isolation of total RNA was performed with strict adherence to the manufacturer’s instructions. RNA concentrations were measured using a NanoDrop ND-1000 Spectrophotometer (Thermo Scientific, Wilmington, DE, United States).

Identical amounts of RNA (1 μg) were reverse – transcribed into cDNA using a commercial RT-PCR kit (Applied Biosystems, Foster City, CA, United States). All reactions were run under the conditions recommended by the manufacturer (total reaction volume 20 μl). The cDNAs were subsequently amplified using PCR Master Mix (Applied Biosystems, Foster City, CA, United States) and TaqMan probes and primers for the following genes: CX3CL1 (Rn00593186_m1), CX3CR1 (Rn00591798_m1), CXCL12 (Rn00573260_m1), CXCR4 (Rn01483207_m1), and CXCR7 (Rn00584358_ m1). Amplification was performed using a 20 μl mixture containing PCR Master Mix, the cDNA used as the PCR template, TaqMan forward and reverse primers and 250 nM of a hydrolysis probe labeled at the 5′-end with the fluorescent reporter FAM and at the 3′-end with a quenching dye. The thermal cycling conditions were 2 min at 50°C and 10 min at 95°C, followed by 40 cycles at 95°C for 15 s and 60°C for 1 min. The threshold value (Ct) for each sample was set during the exponential phase of the PCR, and the ΔΔCt method was used for data analysis. The expression levels were normalized to the Ct for beta-2 microglobulin (b2m) (Rn00560865_m1) as a reference gene.

### Enzyme-Linked Immunosorbent Assay (ELISA)

The tissue samples were homogenized in PBS buffer (Sigma–Aldrich, St. Louis, MO, United States) containing protease inhibitor cocktail (Sigma–Aldrich, St. Louis, MO, United States), phosphatase inhibitor cocktail (Sigma–Aldrich, St. Louis, MO, United States), 1 mM sodium orthovanadate (Sigma–Aldrich, St. Louis, MO, United States), and 1 mM phenylmethanesulfonyl fluoride (Sigma–Aldrich, St. Louis, MO, United States). The lysates were shaken in an ice bath for 30 min and cleared by centrifugation (14,000 rpm, 4°C, 30 min).

The levels of CX3CL1 (fractalkine, Cloud Clone Corporation, Houston, TX, United Stats), CX3CR1 (fractalkine receptor, Cusabio, Washington, DC, United States), SDF-1 (Cloud Clone Corporation, Houston, TX, United States), CXCL12/SDF-1 receptor 4 (CXCR4, MyBiosource, San Diego, CA, United States), CXCL12/SDF-1 receptor 7 (CXCR7, MyBiosource, San Diego, CA, United States) and transforming growth factor β (TGFβ, Cloud Clone Corporation, Houston, TX, United States) in the cortical and hippocampal homogenates were measured using a commercially available enzyme-linked immunosorbent assay (ELISA) kit. Briefly, standards or samples (50 or 100 μl) were pipetted into 96-well plates coated with rat CX3CL1, CX3CR1, CXCL12/SDF-1, CXCR4, and CXCR7 antibodies and incubated. After the plates were extensively washed, HRP-conjugated streptavidin was pipetted into the wells, and the samples were incubated. The wells were washed and 3,3′,5,5′-tetramethylbenzidine (TMB) was added. In this assay, the color develops in proportion to the concentration of the measured protein. The reactions were terminated by the addition of a stop solution. The absorbance was determined using a Tecan Infinite 200 Pro (Tecan, Mannedorf, Germany) system set to the appropriate wavelength (nm). The detection limits were as follows: CX3CL1, 0.054 ng/ml; CX3CR1, 23.5 pg/ml; CXCL12/SDF-1, 6.5 pg/ml; CXCR4, 1 pg/ml; CXCR7, 0.1 ng/ml; and TGFβ, 5.5 pg/ml. Inter-assay precision was as follows: CX3CL1 < 12%, CX3CR1 < 10%, CXCL12/SDF-1 < 12%, CXCR4 < 10%, CXCR7 < 10%, and TGFβ < 12%. Intra-assay precision was as follows: CX3CL1 < 10%, CX3CR1 < 8%, CXCL12/SDF-1 < 10%, CXCR4 < 8%, CXCR7 < 8%, and TGFβ < 10%. Positive controls for each assay were provided by the manufacturers.

### Western Blot

The tissue samples were homogenized in 2% SDS buffer (Sigma–Aldrich, St. Louis, MO, United States). Samples containing equal amounts of protein were heated at 95°C for 5 min in 4x Laemmli sample buffer (Bio-Rad, Hercules, CA, United States). Next, proteins were separated by SDS-PAGE (4–20% gel) under constant voltage (200 V) and were electrophoretically transferred to PVDF membranes (Trans-Blot Turbo; Bio-Rad, Hercules, CA, United States). The blots were blocked in 5% blocking buffer (5% bovine serum albumin) for 1 h at room temperature (RT) and incubated overnight at 4°C with the following primary antibodies that had been diluted in a SignalBoost Immunoreaction Enhancer Kit (Millipore, Warsaw, Poland): anti- Smad2/3 (3102, Cell Signaling, Danvers, MA, United States), anti-phospho-Smad2/3 (8828, Cell Signaling, Danvers, MA, United States), anti-Smad4 (9515, Cell Signaling, Danvers, MA, United States), anti-Smad7 (TA322546, OriGene, Rockville, MD, United States), anti-TGFβr2 (TA347477, OriGene, Rockville, MD, United States), and anti-TGFβr1 (AP01457PU-N, Acris Antibodies, Herfold, Germany). The blots were then incubated at RT with one of the following peroxidase-conjugated secondary antibodies: goat anti-rabbit IgG HRP (PI 1000, Vector Laboratories, Peterborough, United Kingdom) or horse anti-mouse IgG HRP (PI-2000, Vector Laboratories, Peterborough, United Kingdom) for 1–2 h. The immune complexes were detected using Pierce^®^ ECL Western Blotting Substrate (Thermo Fisher, Pierce Biotechnology, Carlsbad, CA, United States) and visualized using a Fujifilm LAS-1000 System (Fuji Film, Tokyo, Japan). The blots were washed two times for 5 min each in TBS; stripped using stripping buffer containing 100 μl of Tris-HCl (pH = 6.7), 2% SDS and 700 μl of 2-mercaptoethanol (all from Sigma–Aldrich, St. Louis, MO, United States); washed two additional times for 5 min each in TBS; blocked; and reprobed with an antibody against GAPDH (MAB374, Millipore, Warsaw, Poland) as an internal loading control at a dilution of 1:5000 in a SignalBoost Immunoreaction Enhancer Kit. All membranes were stripped twice. The relative levels of immunoreactivity were densitometrically quantified using Fujifilm Multi Gauge software (Fuji Film, Tokyo, Japan).

### Statistical Analysis

The outcomes of the behavioral studies are presented as the means ± SEM. The data obtained in the ELISA study are presented as weight units (pg or ng) per milligram of protein ± SEM; those for RT-PCR are presented as the average fold ± SEM, and for Western blot analysis, the results are presented as percentage of the control ± SEM. The normality of variable distribution and homogeneity of variances were checked by Shapiro–Wilk test and Levene’s test, respectively. The significance of the differences between the means was evaluated by one- or two-way analysis of variance (ANOVA), with Duncan’s *post hoc* test if appropriate. When the assumptions of ANOVA were not fulfilled, Kruskal–Wallis ANOVA (by ranks) for multiple comparisons was used. A value of *p* < 0.05 was considered statistically significant. All of the statistical analyses were performed using Statistica software, version 10.0 (Statsoft, Tulsa, OK, United States).

## Results

### The Impact of Prenatal Stress and Chronic Antidepressant Drug Administration on the Immobility, Swimming, and Climbing Times of the Forced Swim Test

Consistent with previous reports (Budziszewska et al., 2010; Głombik et al., 2016), the prenatal stress procedure significantly prolonged immobility time in the FST (*F*_1,57_ = 100.65; *p* < 0.05; **Table 1**). Moreover, when compared to control animals, adult rats exposed to the prenatal stress procedure exhibited a reduction in swimming (*F*_1,57_ = 100.66; *p* < 0.05; **Table 1**), as well as climbing time (*F*_1,57_ = 54,56; *p* < 0.05; **Table 1**), indicating depressive-like behavior.

**Table 1:** The effect of prenatal stress on the times for immobility, swimming, and climbing in the forced swim test.

Forced swim test		
	Control	Prenatal stress
Immobility [s]	192.5 ± 26.9	252.55 ± 18.02^∗^
Swimming [s]	107.5 ± 26.9	47.44 ± 18.02^∗^
Climbing [s]	92.13 ± 30.63	43.44 ± 18.22^∗^

*The results are presented as the mean ± SEM, ^∗^*p* < 0.05 in comparison to control group, *n* = 24–26 for each group. Statistics: one-way ANOVA*.

**FIGURE 2:**
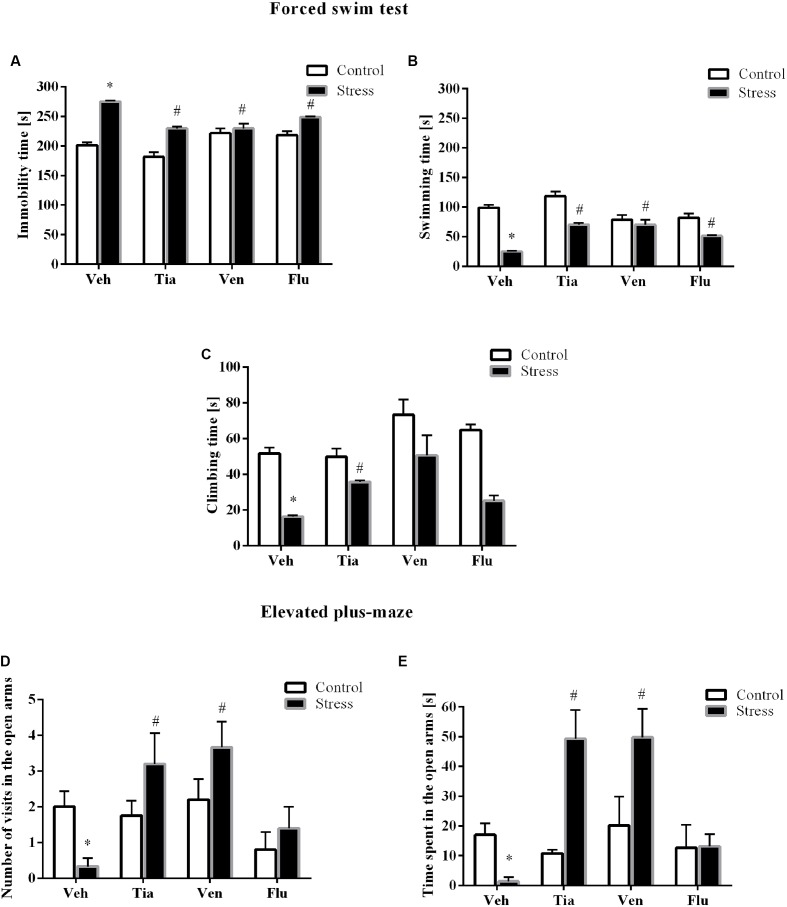
The effects of prenatal stress and chronic antidepressant drug treatment [tianeptine (Tia), venlafaxine (Ven), or fluoxetine (Flu)] on the immobility **(A)**, mobility **(B)**, and climbing time **(C)** (in seconds) in the forced swim test. The effects of prenatal stress and chronic antidepressant drug treatment [tianeptine (Tia), venlafaxine (Ven), or fluoxetine (Flu)] on the number of visits **(D)** and the time spent **(E)** in the open arms of the elevated plus-maze. The data are presented as the means ± SEMs, with *n* = 5–6 for each group. ^∗^*p* ≤ 0.05 vs. control Veh group; ^#^*p* ≤ 0.05 vs. prenatally stressed Veh group. ANOVA (two-way), followed by Duncan’s test or Kruskal–Wallis ANOVA (by ranks) for multiple comparisons.

Next, to determine whether chronic tianeptine, venlafaxine, or fluoxetine administration affected the behavioral changes evoked by prenatal stress, we assessed the rats during the FST again. As we previously demonstrated, enhanced immobility time (*p* < 0.05), shortened swimming (*p* < 0.05), and climbing (*p* < 0.05) were detected in prenatally stressed offspring compared with control offspring. Furthermore, we revealed a significant effect of drugs (*F*_3,39_ = 13.02; *p* < 0.05; **Figure 2A**) on the immobility time. *Post hoc* comparisons revealed that tianeptine (*p* < 0.05), venlafaxine (*p* < 0.05), and fluoxetine (*p* < 0.05) shortened immobility time in prenatally stressed offspring. We also observed a significant effect of the drugs (*F*_3,39_ = 13.02; *p* < 0.05; **Figure 2B**) on swimming time. Moreover, *post hoc* comparisons revealed that tianeptine (*p* < 0.05), venlafaxine (*p* < 0.05), and fluoxetine (*p* < 0.05) extended the swimming time in stressed offspring. Regarding climbing time (**Figure 2C**), we observed that only tianeptine (*F*_3,38_ = 7.94; *p* < 0.05) prolonged the climbing time in prenatally stressed rats.

### The Impact of Prenatal Stress and Chronic Antidepressant Drug Administration on Anxiety-Like Behavior in the Elevated Plus-Maze Test

To assess anxiety-like behavior in adult rats, we performed the elevated plus-maze test. As we previously demonstrated (Głombik et al., 2017), the prenatal stress procedure leads to a significant reduction in the number of entries into the open arms (*F*_1,34_ = 32.46; *p* < 0.05; **Figure 2D**) of the maze and a significant decrease in the time spent in them (*F*_1,34_ = 88.57; *p* < 0.05; **Figure 2E**). However, we did not observe differences in the number of entries into the closed arms and the time spent in them (data not shown).

*Post hoc* comparisons showed that tianeptine (*p* < 0.05) and venlafaxine (*p* < 0.05) enhanced the number of entries into the open arms of the maze (*p* < 0.05) and the time spent in them (*p* < 0.05).

### The Impact of Prenatal Stress and Chronic Antidepressant Drug Administration on the mRNA Expression and Protein Levels of CX3CL1 and Its Receptor CX3CR1 in the Hippocampus and the Frontal Cortex of Adult Offspring Rats

**FIGURE 3:**
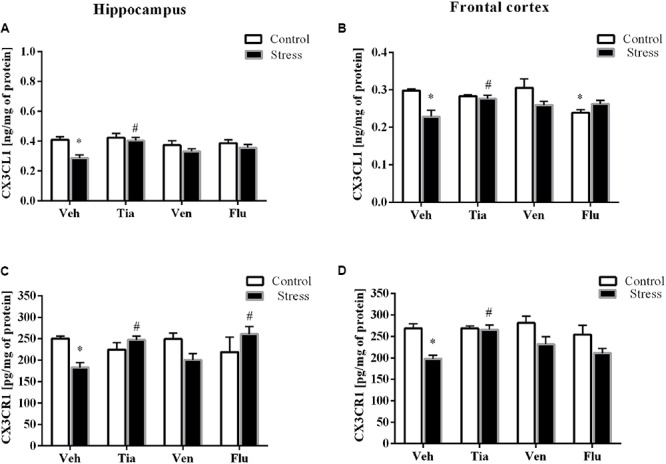
The effect of prenatal stress and antidepressant drugs treatment [tianeptine (Tia), venlafaxine (Ven), or fluoxetine (Flu)] on CX3CL1 level (ng/mg of protein) in the hippocampus **(A)** and frontal cortex **(B)** and on the level of its receptor – CX3CR1 (pg/mg of protein) in these brain areas **(C,D)**. The data are presented as the means ± SEM, with *n* = 5–6 for each group. ^∗^*p* < 0.05 vs. control Veh group; ^#^*p* < 0.05 vs. prenatally stressed Veh group. ANOVA (two-way), followed by Duncan’s test.

In the present study, the analyses of tissue samples revealed that the prenatal stress procedure did not affect the gene expression of CX3CL1 (**Table 2A**) in the hippocampus. However, in the frontal cortex of prenatally stressed offspring, the mRNA expression of CX3CL1 was significantly diminished (*F*_1,39_ = 5.97; *p* < 0.05). Chronic administration of tianeptine (*p* < 0.05) and fluoxetine (*p* < 0.05) normalized the prenatal stress-induced changes in CX3CL1 expression in the frontal cortex (**Table 2B**). On the other hand, ELISA revealed significantly diminished protein levels of CX3CL1 in both the hippocampus (*F*_1,36_ = 9.67; *p* < 0.05; **Figure 3A**) and the frontal cortex (*F*_1,36_ = 7.66; *p* < 0.05; **Figure 3B**). Only chronic administration of tianeptine normalized the changes in CX3CL1 levels caused by prenatal stress in both brain areas (*p* < 0.05; **Figures 3A,B**).

**Table 2:** The effect of prenatal stress (PS) and antidepressant drugs treatment [tianeptine (Tia), venlafaxine (Ven), or fluoxetine (Flu)] on the mRNA expression of chemokine and chemokine receptors in the hippocampus **(A)** and frontal cortex **(B)** of adult rats.

A (Gene expression hippocampus)								
Factor	Control	PS	Control+TIA	PS+TIA	Control+VEN	PS+VEN	Control+FLU	PS+FLU
CX3CL1	0.91 ± 0.01	0.78 ± 0.04	0.91 ± 0.02	0.98 ± 0.05	1.07 ± 0.03	0.83 ± 0.02	1.01 ± 0.04	0.90 ± 0.07
CX3CR1	0.97 ± 0.03	**0.77 ± 0.06^∗^**	0.92 ± 0.02	**0.97 ± 0.04^#^**	1.10 ± 0.07	0.80 ± 0.06	0.99 ± 0.06	0.80 ± 0.03
CXCL12	0.93 ± 0.03	**1.18 ± 0.03^∗^**	0.97 ± 0.04	**0.94 ± 0.03^#^**	0.93 ± 0.05	1.20 ± 0.02	1.08 ± 0.06	1.30 ± 0.03
CXCR4	1.00 ± 0.01	**0.74 ± 0.02^∗^**	0.99 ± 0.02	**1.00 ± 0.05^#^**	1.06 ± 0.01	**1.00 ± 0.01^#^**	1.01 ± 0.04	0.81 ± 0.06
CXCR7	1.00 ± 0.03	**1.38 ± 0.04^∗^**	1.04 ± 0.04	**0.98 ± 0.03^#^**	0.97 ± 0.02	1.24 ± 0.05	0.99 ± 0.04	1.40 ± 0.08

**B** **(Gene expression frontal cortex)**								
CX3CL1	1.00 ± 0.02	**0.81 ± 0.02^∗^**	0.92 ± 0.02	**0.99 ± 0.05^#^**	0.93 ± 0.02	0.83 ± 0.05	1.02 ± 0.04	**0.98 ± 0.04^#^**
CX3CR1	1.00 ± 0.03	**0.71 ± 0.04^∗^**	0.94 ± 0.04	**0.93 ± 0.04^#^**	0.90 ± 0.01	0.84 ± 0.04	0.68 ± 0.11	0.64 ± 0.09
CXCL12	1.00 ± 0.03	0.91 ± 0.05	1.03 ± 0.04	0.92 ± 0.07	1.04 ± 0.05	1.14 ± 0.07	0.93 ± 0.05	0.91 ± 0.07
CXCR4	1.00 ± 0.03	**0.83 ± 0.02^∗^**	0.99 ± 0.05	0.96 ± 0.03	1.06 ± 0.04	0.92 ± 0.05	1.02 ± 0.06	0.81 ± 0.04
CXCR7	1.00 ± 0.03	**1.30 ± 0.08^∗^**	0.94 ± 0.02	**0.95 ± 0.04^#^**	1.02 ± 0.03	1.28 ± 0.03	0.99 ± 0.05	1.28 ± 0.05

*Results are expressed as average fold ± SEM. The number of animals in each group: *n* = 5–6. ^∗^*p* ≤ 0.05 vs. control Veh group; ^#^*p* ≤ 0.05 vs. prenatally stressed Veh group Statistics: two-way ANOVA followed by Duncan test. When the assumptions of ANOVA were not fulfilled, Kruskal–Wallis ANOVA (by ranks) for multiple comparisons was used. *p* < 0.05. Bolded values are statistically significant*.

In the next set of experiments, analyses of hippocampal (*F*_1,33_ = 19.28; *p* < 0.05) and cortico-frontal (*F*_1,38_ = 4.92; *p* < 0.05) samples revealed a significant down-regulation of the mRNA expression of CX3CR1 in prenatally stressed rats compared with control animals. Chronic treatment with tianeptine normalized the gene expression changes in both brain areas (*p* < 0.05; **Tables 2A,B**). Furthermore, we demonstrated that the prenatal stress procedure-induced diminished protein levels of CX3CR1 in the hippocampus (*F*_1,35_ = 0.59; *p* < 0.05; **Figure 3C**) and the frontal cortex (*F*_1,39_ = 18.20; *p* < 0.05; **Figure 3D**) were normalized by tianeptine treatment (*p* < 0.05). Moreover, fluoxetine (*p* < 0.05) attenuated the changes in CX3CR1 protein levels caused by prenatal stress in the hippocampus (**Figure 3C**).

### The Impact of Prenatal Stress and Chronic Antidepressant Drug Administration on the mRNA Expression and Protein Levels of CXCL12 and Its Receptors: CXCR4 and CXCR7 in the Hippocampus and the Frontal Cortex of Adult Offspring Rats

**FIGURE 4:**
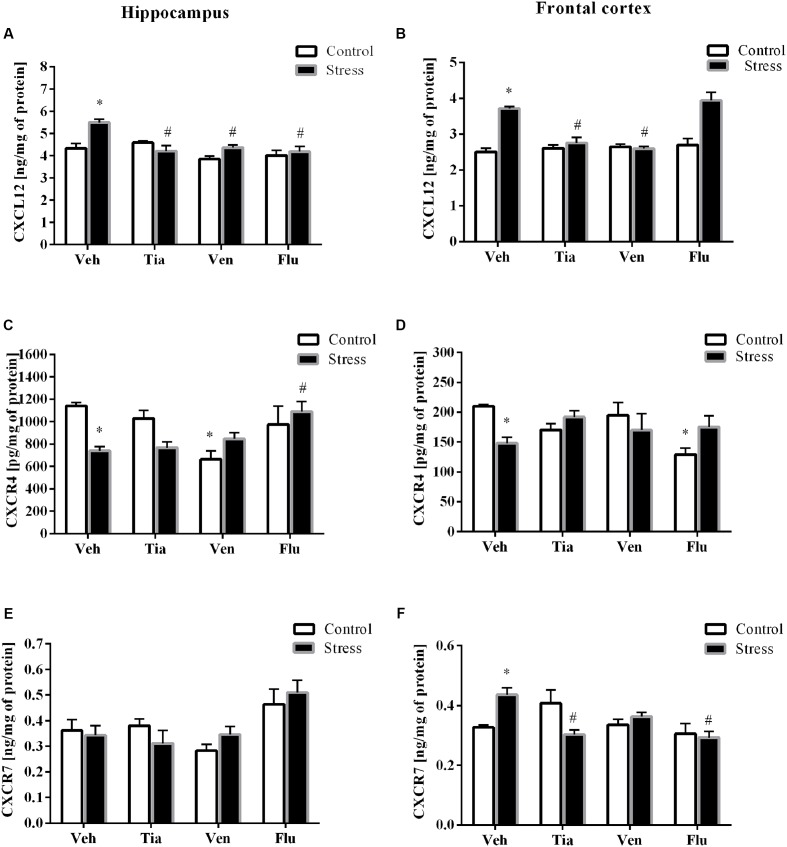
The effect of prenatal stress and antidepressant drugs treatment [tianeptine (Tia), venlafaxine (Ven), or fluoxetine (Flu)] on CXCL12 level (ng/mg of protein) in the hippocampus **(A)** and frontal cortex **(B)** and on the level of receptors – CXCR4 (pg/mg of protein; **C,D**) and CXCR7 (ng/mg of protein; **E,F**) also in these brain areas The data are presented as the means ± SEM, with *n* = 5–6 for each group. ^∗^*p* < 0.05 vs. control Veh group; ^#^*p* < 0.05 vs. prenatally stressed Veh group. ANOVA (two-way), followed by Duncan’s test.

The results of ANOVA showed a significant increase in CXCL12 expression in the hippocampus (*F*_1,33_ = 35.26; *p* < 0.05) of prenatally stressed offspring (**Table 2A**). Chronic administration of tianeptine (*p* < 0.05) normalized these changes. As shown in **Figure 4**, we observed statistically significant up-regulation of CXCL12 levels in the hippocampus (*F*_1,38_ = 41.15; *p* < 0.05; **Figure 4A**) and the frontal cortex (*F*_1,37_ = 40.67; *p* < 0.05; **Figure 4B**). Chronic administration of tianeptine (*p* < 0.05), venlafaxine (*p* < 0.05), and fluoxetine (*p* < 0.05) in the hippocampus, while tianeptine (*p* < 0.05) and venlafaxine in the frontal cortex (*p* < 0.05) normalized the changes in CXCL12 levels caused by prenatal stress (**Figures 4A,B**).

In the next set of experiments, we reported a significant decrease in the gene expression levels of CXCR4 in the hippocampus (*F*_1,39_ = 28.59; *p* < 0.05) and the frontal cortex (*F*_1,38_ = 20.39; *p* < 0.05; **Tables 2A,B**). Chronic administration of tianeptine (*p* < 0.05) and venlafaxine (*p* < 0.05) normalized these changes only in the hippocampus. Our experiments showed that the prenatal stress procedure diminished the CXCR4 concentration in the hippocampus (*F*_1,38_ = 2.42; *p* < 0.05; **Figure 4C**) and the frontal cortex (*F*_1,39_ = 0.17; *p* < 0.05; **Figure 4D**). We observed the impact of venlafaxine (*p* < 0.05) in the hippocampus and the effect of fluoxetine (*p* < 0.05) in the frontal cortex on the CXCR4 concentration in control animals. Among the tested antidepressants, *post hoc* comparison found that only fluoxetine treatment enhanced CXCR4 levels in the hippocampus of prenatally stressed offspring (*p* < 0.05; **Figure 4C**).

Since CXCL12 also exerts biological activity *via* CXCR7, we therefore examined the gene expression and protein levels of CXCR7 in both the hippocampus and the frontal cortex. A significant increase in CXCR7 expression levels in the hippocampus (*F*_1,38_ = 68.25; *p* < 0.05) and the frontal cortex (*F*_1,39_ = 45.81; *p* < 0.05) of prenatally stressed offspring was observed, which was attenuated only by chronic tianeptine treatment (*p* < 0.05; **Tables 2A,B**).

Furthermore, our study demonstrated that neither prenatal stress nor chronic antidepressant administration affected CXCR7 concentrations in the hippocampus (**Figure 4E**). In contrast, prenatally stressed rats exhibited significantly increased levels of CXCR7 in the frontal cortex (*F*_1,47_ = 1.08; *p* < 0.05), while chronic tianeptine (*p* < 0.05) or fluoxetine (*p* < 0.05) administration normalized this parameter (**Figure 4F**).

### The Impact of Prenatal Stress and Chronic Antidepressant Drug Administration on the Protein Levels of TGFβ and Both Receptors, TGFβr1 and TGFβr2, in the Hippocampus and the Frontal Cortex of Adult Offspring Rats

**FIGURE 5:**
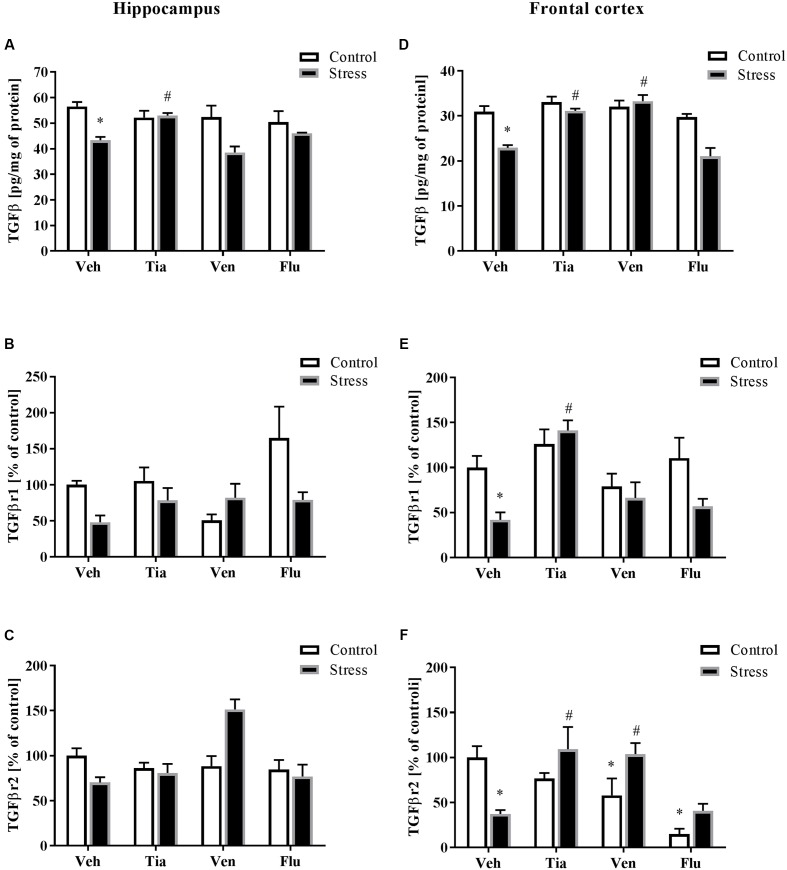
The effect of prenatal stress and antidepressant drugs treatment [tianeptine (Tia), venlafaxine (Ven), or fluoxetine (Flu)] on the protein levels of TGFβ (pg/mg of protein), TGFβR1 (% of control), and TGFβR2 (% of control) in the hippocampus **(A–C)** and frontal cortex **(D–F)**. The data are presented as the means ± SEM, with *n* = 5–6 for each group. ^∗^*p* < 0.05 vs. control Veh group; ^#^*p* < 0.05 vs. prenatally stressed Veh group. ANOVA (two-way), followed by Duncan’s test (Supplementary Data Sheet 1).

As reported previously, the malfunction in the TGFβ pathway may play an important role in the regulation of the brain CX3CL1–CX3CR1 axis (Chen et al., 2002). Therefore, we examined the effect of the prenatal stress procedure and chronic antidepressant drug administration on TGFβ, TGFβr1, and TGFβr2 levels in the hippocampus and the frontal cortex. Regarding the differences between control and prenatally stressed rats, a significant decrease in TGFβ protein levels in the hippocampus (*F*_1,37_ = 13.68; *p* < 0.05) and the frontal cortex (*F*_1,37_ = 25.56; *p* < 0.05) was observed (**Figures 5A,D**). Moreover, we demonstrated that only tianeptine (*p* < 0.05) ameliorated prenatal stress-induced reductions in TGFβ levels in the hippocampus and frontal cortex (**Figures 5A,D**), while venlafaxine (*p* < 0.05) administration only in the frontal cortex (**Figure 5D**).

Next, we showed that neither prenatal stress nor antidepressant treatment affected the levels of TGFβr1 or TGFβr2 in the hippocampus of adult offspring (**Figures 5B,C**). In contrast, in **Figures 5E,F**, we demonstrated significant down-regulation of TGFβr1 (*F*_1,30_ = 5.75; *p* < 0.05) and TGFβr2 (*F*_1,26_ = 2.20; *p* < 0.05) in the frontal cortex of adult offspring after prenatal stress. Tianeptine (*p* < 0.05) treatment normalized changes in both TGFβr1 (**Figure 5E**) and TGFβr2 concentrations (**Figure 5F**), while venlafaxine (*p* < 0.05) only normalized changes in TGFβr2. Additionally, our study found that venlafaxine and fluoxetine diminished TGFβr2 levels in control rats (**Figure 5F**) (Supplementary Data Sheet 1).

### The Impact of Prenatal Stress and Chronic Antidepressant Drug Administration on the Canonical TGFβ Receptors Pathways in the Hippocampus and the Frontal Cortex of Adult Offspring Rats

**FIGURE 6:**
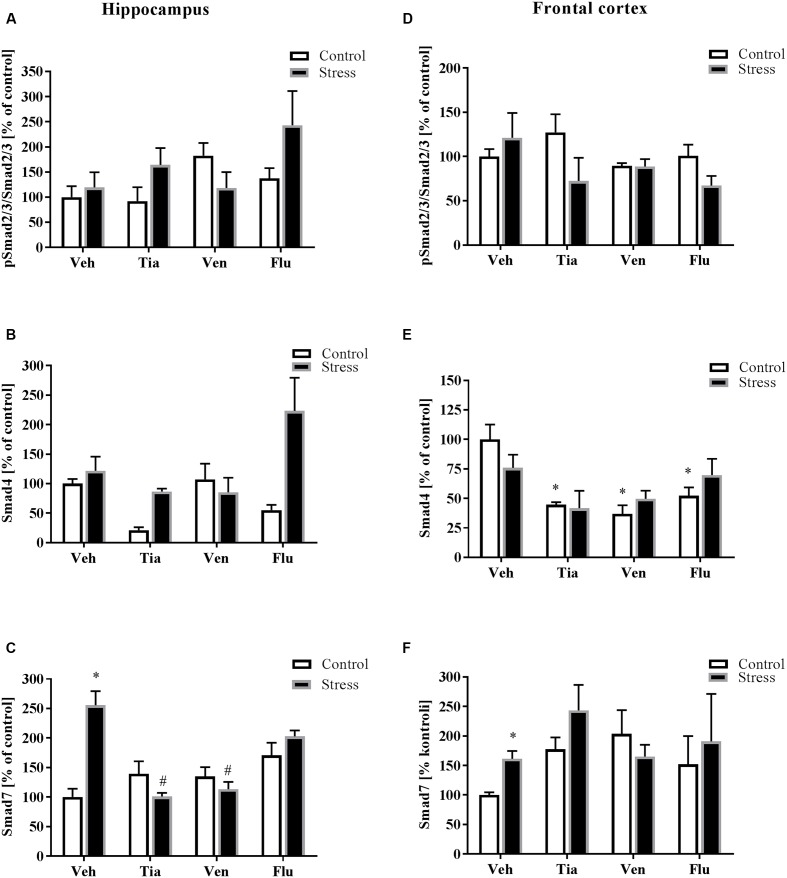
The effect of prenatal stress and antidepressant drugs treatment [tianeptine (Tia), venlafaxine (Ven), or fluoxetine (Flu)] on the activation of Smad2/3 pathway in the hippocampus **(A–C)** and in the frontal cortex **(D–F)**. The data are presented as % of control, with *n* = 5–6 for each group. ^∗^*p* < 0.05 vs. control Veh group; ^#^*p* < 0.05 vs. prenatally stressed Veh group. ANOVA (two-way), followed by Duncan’s test (Supplementary Data Sheet 1).

Since data demonstrated that Smad signaling is essential for TGFβ gene responses, we measured the impact of the prenatal stress procedure and antidepressant administration on the level of the phosphorylated active form of Smad2/3 (pSmad2/3/Smad2/3) as well as protein levels of Smad4 and Smad7 (**Figure 6**). We found that prenatal stress did not affect pSmad2/3/Smad2/3 and Smad4 levels in the hippocampus of adult prenatally stressed animals (**Figures 6A,B**). However, the level of Smad7 in this structure in prenatally stressed offspring was significantly up-regulated (*F*_1,28_ = 5.42; *p* < 0.05; **Figure 6C**) and normalized by chronic tianeptine (*p* < 0.05) or venlafaxine (*p* < 0.05) administration (**Figure 6C**). In the frontal cortex, we did not observe the impact of either prenatal stress or antidepressant drug treatment on pSmad2/3/Smad2/3 (**Figure 6D**) and Smad4 (**Figure 6E**) levels. However, in control offspring, the down-regulation of Smad4 was noted following treatment with each antidepressant. Importantly, as in the hippocampus, the level of Smad7 in the frontal cortex of prenatally stressed offspring was up-regulated (*F*_1,35_ = 10.96; *p* < 0.05; **Figure 6F**) (Supplementary Data Sheet 1).

## Discussion

Prenatal stress is a well-recognized animal model of depression. The usefulness of this procedure as a model of depression has been verified by reports of compliance with the requirements for construct, face and predictive validity (Morley-Fletcher et al., 2003). Our study confirmed that behavioral deficits are present in the offspring of rat dams that were stressed during the last week of pregnancy (Trojan et al., 2016; Głombik et al., 2017). Moreover, prolonged immobility and diminished swimming time were normalized by chronic tianeptine, fluoxetine, or venlafaxine treatment. Additionally, tianeptine administration attenuated the deficits in climbing behavior caused by stress. Tianeptine and venlafaxine attenuated anxiety-like behavior evoked by prenatal stress, as assessed by an increase in the number of entries and in the time spent in the open arms of the maze.

Our study demonstrated that chronic treatment with antidepressants, in addition to the beneficial impact on the behavioral alterations, attenuated in drug-dependent manner the malfunction evoked by prenatal stress in the chemokine – chemokine receptor network in the hippocampus and the frontal cortex of adult offspring. Considering that CXCL12, CX3CL1 and their receptors play a crucial role in brain homeostasis and the course of pathological conditions, e.g., neuroinflammation, disturbances in these molecules expression, have been proposed as a potential background of brain disorders, including depression.

In the present study, we clearly demonstrated that prenatal stress increases CXCL12 levels in the hippocampus and the frontal cortex of adult offspring. Importantly, elevated CXCL12 levels in the hippocampus were normalized by chronic administration of all tested antidepressants, while levels in the frontal cortex by tianeptine and venlafaxine treatment.

Data show that CXCL12 is expressed under homeostatic conditions, whereas its expression is strongly up-regulated during inflammation, hypoxia or ischemia. However, recent data showed that CXCL12 may also have an anti-inflammatory property that mediates immune cell recruitment, which leads to limited inflammation. Thus far, only some experimental data demonstrated CXCL12 changes in the brain in stress-related model (Ślusarczyk et al., 2015). Interestingly, a potential GABA-mediated inhibitory role of secreted CXCL12 on the serotoninergic system, which may lead to depressive behavior, has been observed (Réaux-Le Goazigo et al., 2013). In line with this finding, an increase in the levels of CXCL12 and RANTES has been observed in patients with major depression, and suppression of these molecules has been found following treatment with antidepressant drugs (e.g., fluoxetine) (Shen et al., 2010). Notably, enhanced brain CXCL12 levels may also potentiate TNF-α release that not only causes production of glutamate, which directly affects astrocytes, but also leads to activation of receptors on microglia. Consequently, enhanced TNF-α production can lead to neurotoxicity (Calì et al., 2008, 2010). Therefore, in our study, the normalizing impact of antidepressants on CXCL12 levels in the studied brain areas may have a broad, indirect anti-inflammatory value.

CXCL12 is well-known to mediate biological function in brain through the two following receptors: classical CXCR4 and non-classical CXCR7 (Banisadr et al., 2002, 2003). We observed that prenatal stress diminished the levels of CXCR4 in both examined areas. However, chronic fluoxetine treatment attenuated prenatal stress-induced changes only in the hippocampus. Since CXCR4 modulates neurotransmitter release and hormone secretion from neuroendocrine cells (Bezzi et al., 2001) and is crucial in the modulation of brain inflammation (Schönemeier et al., 2008), the observed malfunction of the CXCR12–CXCR4 axis in the present study may interfere with these processes. The diminished expression levels of CXCR4 may also affect neuronal viability of rat brain cortical neurons, as well as several metabolic parameters (Merino et al., 2016). Interestingly, the results of Felszeghy et al. (2004) showed that dexamethasone down-regulates CXCR4 receptor expression. Therefore, we may postulate that enhanced levels of endogenous glucocorticoids in prenatally stressed rats (Szymańska et al., 2009) may be responsible for decreased CXCR4 brain expression. To date, the mechanisms responsible for the impact of antidepressants on the CXCL12–CXCR4 brain axis have not been studied in any animal model of depression, so further understanding of the fluoxetine signaling pathways downstream of CXCR4 will be crucial.

In contrast to CXCR4 in the present study, we observed enhanced CXCR7 receptor levels, which were normalized by tianeptine or fluoxetine treatment, in the frontal cortex of prenatally stressed rats. CXCR7 does not activate G_αi_ signaling but acts as a β-arrestin-biased receptor (Levoye et al., 2009; Rajagopal et al., 2010). The broad expression of CXCR7 receptor in the brain may suggest CXCR4-independent function of CXCL12 (Banisadr et al., 2016). Therefore, in the present study, prenatal stress procedure-induced down-regulation of CXCR4 and up-regulation of CXCR7 receptors may lead to disparate effects of CXCL12. Our study is the first showing the ability of chronic fluoxetine treatment to normalize the brain CXCL12-CXCR4-CXCR7 axis in an animal model of depression.

The present study demonstrated that among antidepressants tested only chronic tianeptine administration normalized the diminished protein levels of CX3CL1 and CX3CR1 in both the hippocampus and the frontal cortex of adult offspring. Thus far, data concerning the impact of antidepressant drugs on CX3CL1–CX3CR1 brain signaling in animal models of depression are scarce and ambiguous. Treatment with imipramine and agomelatine did not reverse the changes in the dorsal hippocampus evoked by chronic mild stress, while chronic imipramine or lurasidone treatment diminished CX3CR1 mRNA expression in these structures (Rossetti et al., 2016). Recently, the Alboni et al. (2016) group demonstrated that the impact of chronic fluoxetine administration on the CX3CL1–CX3CR1 axis in the hippocampus depends on the quality of the living environment. In stressful conditions, CX3CL1 gene expression was reduced in fluoxetine-treated mice, whereas in enriched conditions, no changes were found. In the case of venlafaxine, its chronic treatment ameliorated depressive-like behavior and restored microglial morphology in control (wide type) animals, whereas resistance to stress-induced depressive-like behavior and changes in microglial morphology after venlafaxine treatment in CX3CR1 deficient mice was recently noted (Hellwig et al., 2016). In the present study, however, venlafaxine had no effect on CX3CR1 and CX3CL1 expression in both brain regions – though its anxiolytic and anti-depressive properties were observed. Therefore, further studies are needed to unequivocally identify the involvement of CXCL1–CXCR1 axis in behavioral effects of this drug.

On the other hand, in our previous functional study, we demonstrated that 7 days after icv CX3CL1 treatment, adult prenatally stressed rats showed an increased swimming and climbing time and a decreased immobility time. Furthermore, CX3CL1 administration reduced stress-induced anxiety-like behavior (Ślusarczyk et al., 2016). Taking into account this observation, we can speculate that, in contrast to venlafaxine, beneficial effect of tianeptine on behavioral disturbances observed in adult offspring may be at least partially related with normalization of the CX3CL1–CX3CR1 axis. In the context of our results, one particularly relevant observation is that proper CX3CL1 and CX3CR1 concentrations are required for maintaining microglia in a “resting state” through modulation of their activity and pro-inflammatory factor release (Cardona et al., 2006; Corona et al., 2010; Frick et al., 2013). Thus, the normalization of the brain CX3CL1–CX3CR1 axis by tianeptine may be the result of its suppressive effect on microglial over-activation and pro-inflammatory factor release (Ślusarczyk et al., 2015), which was demonstrated in our previous study in prenatally stressed offspring. The limitation of our study is the fact that we performed our research only in male offspring, whereas recent data showed sex differences in microglial density, morphology and activation in hippocampus and frontal cortex (Schwarz et al., 2012). Moreover, the sex-dependent expression of CX3CL1–CX3CR1 in brain in basal condition was demonstrated (Bollinger et al., 2016), which might determine not only the susceptibility to stress, the profile of behavioral disturbances but maybe also tianeptine action.

Several studies indicate that TGFβ is an interesting suppressor of microglial cell activation (Suzumura et al., 1993; Lodge and Sriram, 1996; Chen et al., 2002). In this study, we demonstrated diminished concentrations of TGFβ in the hippocampus and the frontal cortex of prenatally stressed rats. Tianeptine treatment enhanced TGFβ levels in both examined areas, while venlafaxine increased levels in the frontal cortex. In line with these results, rats subjected to a chronic mild stress (model of depression) exhibit significant decreases in TGFβ in the brain (You et al., 2011), while depressive patients show a reduction in TGFβ serum levels (Sutcigil et al., 2007; Musil et al., 2011). Interestingly, TGFβ levels showed a significant negative correlation with the Hamilton Depression Rating Scale (HDRS) (Myint et al., 2005), while TGFβ levels increased in depressed patients after 6 weeks of treatment with fluoxetine, venlafaxine, or paroxetine (Lee and Kim, 2006). Because TGFβ signaling is mainly localized to microglia (Kiefer et al., 1995), it is considered the alternative regulator of their activation (Spittau et al., 2013). Specifically, TGFβ increased CX3CR1 and reduced IL-1β expression in activated microglia, leading to resolution of microglial activation and a return to baseline behavior after LPS challenge (Henry et al., 2009; Wynne et al., 2010). Moreover, in aged mice, TGFβ inhibition resulted in an inflammatory phenotype of microglia, including enhanced IL-1β and lower CX3CR1 expression levels. Additionally, the mRNA levels of both TGFβ and CX3CR1 dynamically change in a corresponding time-dependent manner, indicating a relationship between these two protein systems. Because our study found that tianeptine up-regulated TGFβ release, this signaling pathway should be taken into account as a conceivable mechanism of the action of tianeptine on CX3CR1 concentrations in the hippocampus and the frontal cortex of prenatally stressed rats.

In the present study, we examined the canonical TGFβ signaling cascade, which initiates TGF-β binding to the type II transmembrane receptor serine/threonine kinases (TGFβr2) that in turn assemble with, phosphorylate and activate the type I receptor (TGFβr1; ALK5). We observed that prenatal stress diminished TGFβr2 and TGFβr1 levels in the frontal cortex. We also reported that chronic tianeptine treatment normalized the stress-induced malfunction in both TGFβ receptors, while venlafaxine only affected TGFβr2. Activated TGFβr1 may phosphorylate the downstream effectors Smad2 and Smad3, which then associate with Smad4 (Edlund et al., 2003; Bakkebø et al., 2010). Additionally, alternative non-Smad pathways, including ERK1/2, JNK, p38 MAPK and the tyrosine kinase Src or PI3K, can be activated (Moustakas and Heldin, 2005). In our study, neither prenatal stress nor antidepressant treatment affected Smad2/3 phosphorylation, while all tested antidepressants diminished Smad4 levels in control offspring. In contrast to our study, Dow et al. (2005) indicated that chronic antidepressant treatment increases TGFβ-mediated phosphorylation of Smad2 (pSmad2). The lack of impact of antidepressants on pSmad2 in our study may be due to the fact that Smad2 and 3 are also phosphorylated and activated by several other ligands and receptor complexes of the TGFβ family or directly by other kinases (Moustakas and Heldin, 2009). Moreover, other pathways may induce inhibitory Smads (I-Smads), consequently suppressing TGFβ receptor signaling. Among these signaling molecules, TNF-α and IL-1β induce Smad7 (Bitzer et al., 2000; Mitchell et al., 2014). Interestingly, our results showed that prenatal stress up-regulated Smad7 levels in the hippocampus and the frontal cortex and thus exerted an inhibitory effect on TGFβ signaling. Moreover, chronic tianeptine or venlafaxine treatment diminished prenatal stress-induced up-regulation of Smad7 in the hippocampus. In parallel, Smad7 interferes with TGFβ signaling by binding to TGFβr1 to prevent Smad2/3 phosphorylation and activation or recruitment of the protein phosphatase or the ubiquitin ligases to the receptor, leading to either receptor dephosphorylation or proteasomal degradation (Koinuma et al., 2003; Kamiya et al., 2010).

Taken together, the outcomes of the present study show that prenatal stress leads to anxiety and depressive-like disturbances in adult animals. Moreover, for the first time, we demonstrated that the evoked by prenatal stress procedure dysfunctions of constitutively expressed brain chemokines, CXCL12, and their receptors and at less extend CX3CL1 and CX3CR1, in hippocampus and frontal cortex of adult offspring, were attenuated by chronic antidepressant drug treatment. This drug-dependent action seems to be bi-directional and manifests as inhibition of pro-inflammatory CXCL12 expression and partially stimulation of anti-inflammatory CX3CL1 and TGFβ release.

## Author Contributions

AB-K, ET, and JŚ were responsible for the conception and design of the study. ET, JŚ, KG, and KK performed behavioral analyses. ET, JŚ, KC, and KK were responsible for biochemical analyses of the samples. ET was responsible for the interpretation of the data. ET and JŚ drafted the article. MK helped to write the final version of the manuscript. All authors revised the paper critically for important intellectual content and gave final approval of the version to be published.

## Conflict of Interest Statement

The authors declare that the research was conducted in the absence of any commercial or financial relationships that could be construed as a potential conflict of interest.
