# Supplementary material for: The Modulatory Properties of Chronic Antidepressant Drugs Treatment on the Brain Chemokine – Chemokine Receptor Network: A Molecular Study in an Animal Model of Depression
Source: Front Pharmacol. 2017 Nov 1;8:779. doi: 10.3389/fphar.2017.00779 (PMC5671972; doi:10.3389/fphar.2017.00779)
Supplement: Supplementary file 1 [file Data_Sheet_1.PDF]

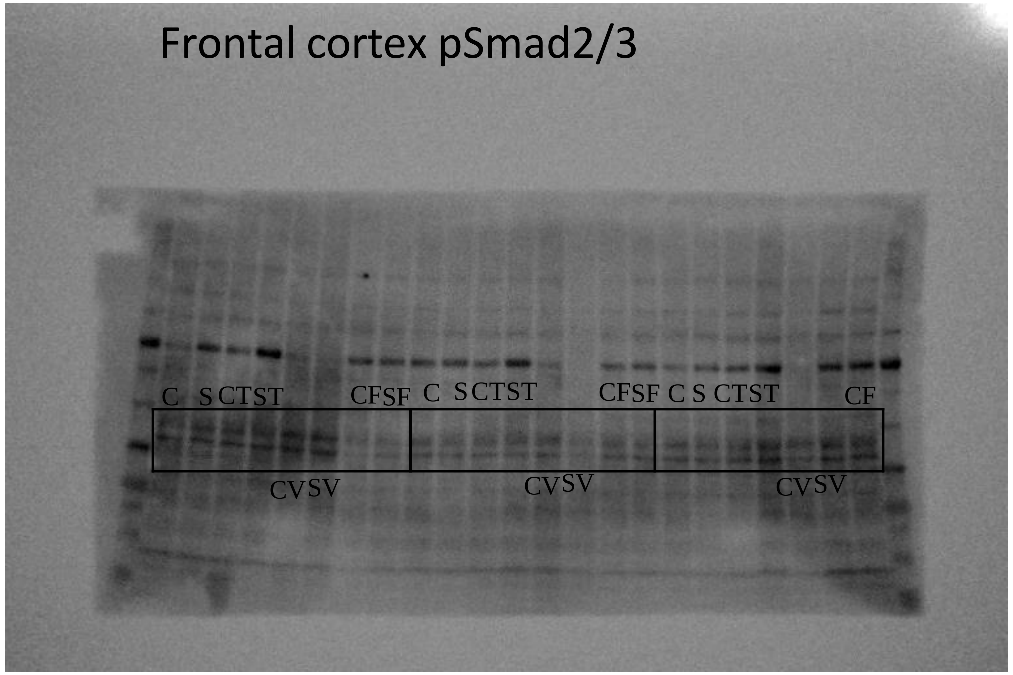

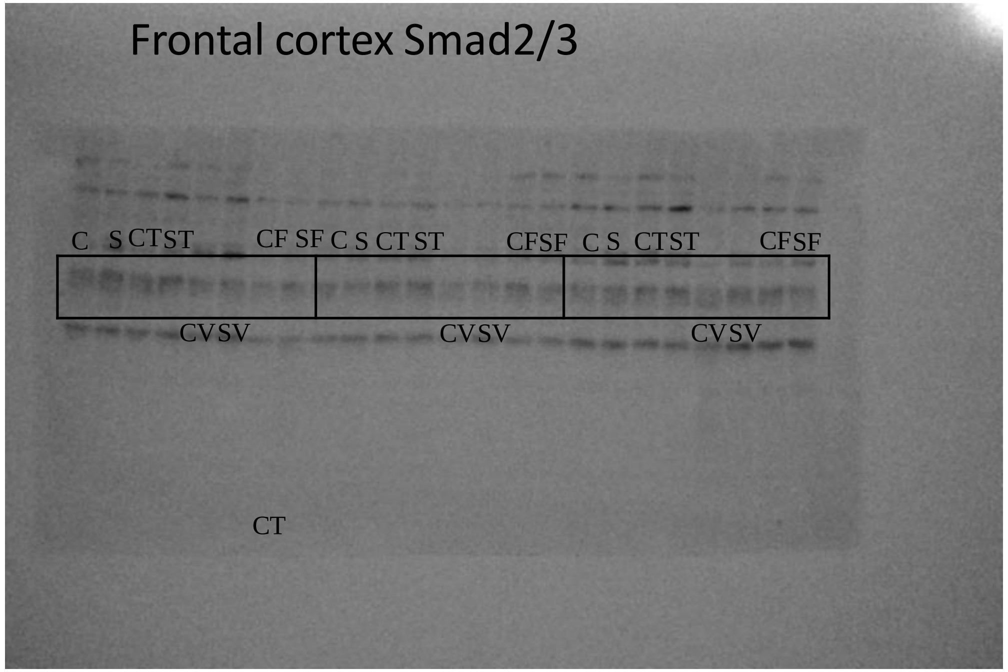

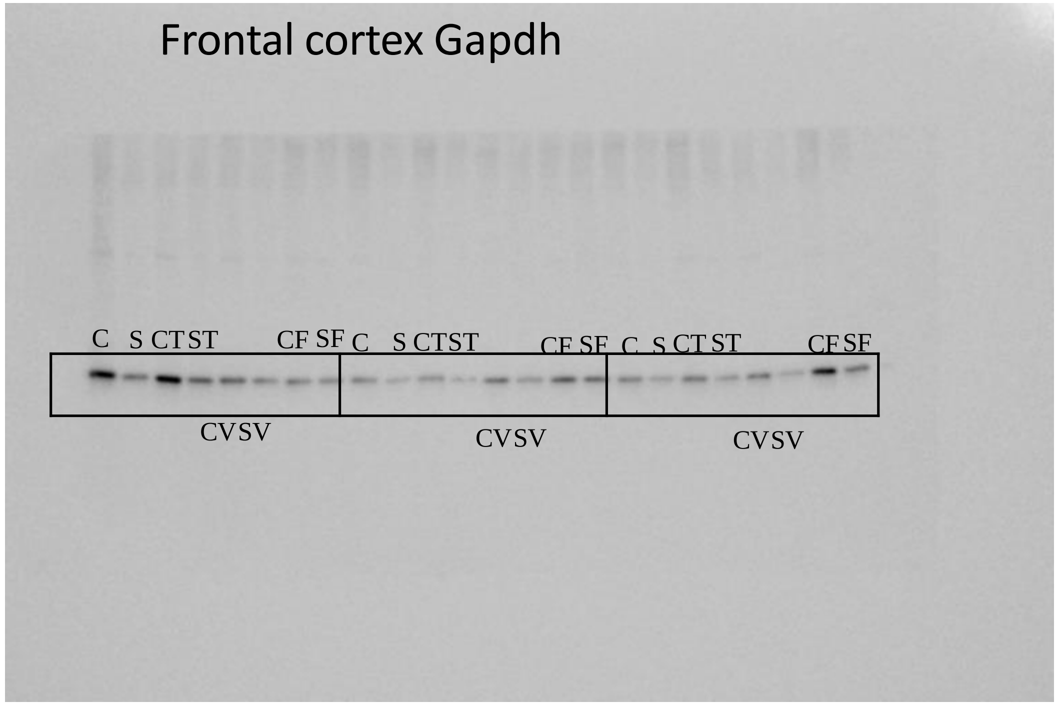

C – CONTROL+VEH
S – STRESS+VEH
CT - CONTROL+TIA
ST – STRESS+TIA
CV – CONTROL+VEN
SV – STRESS+VEN
CF – CONTROL+FLU
SF – STRESS+FLU

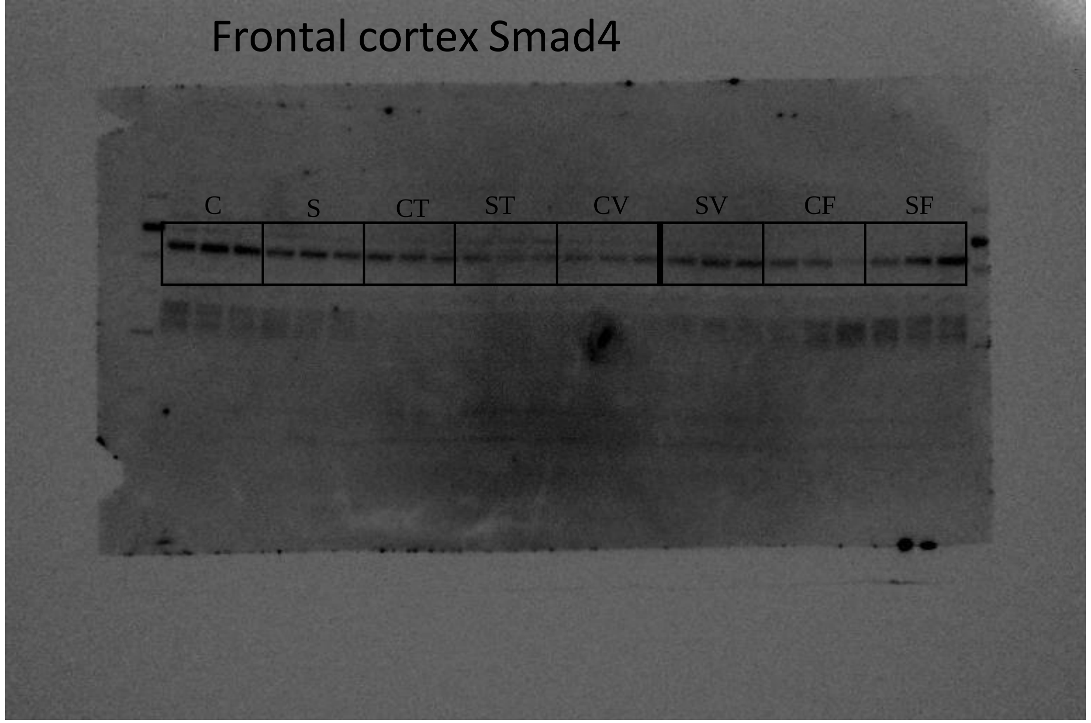

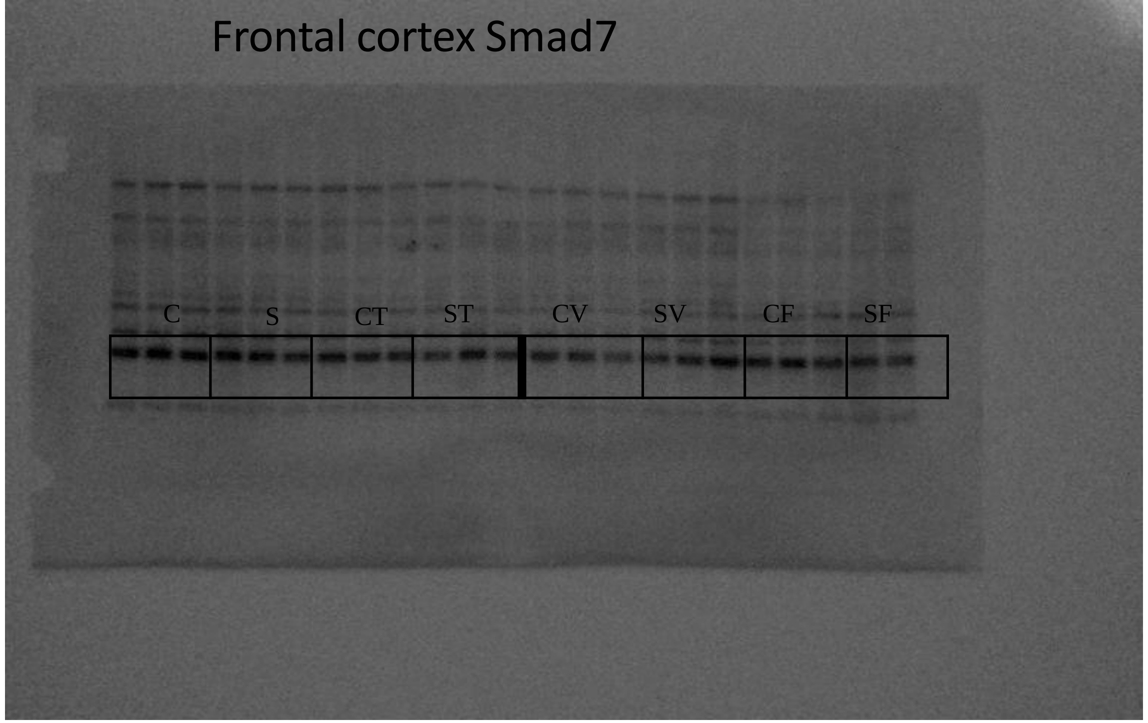

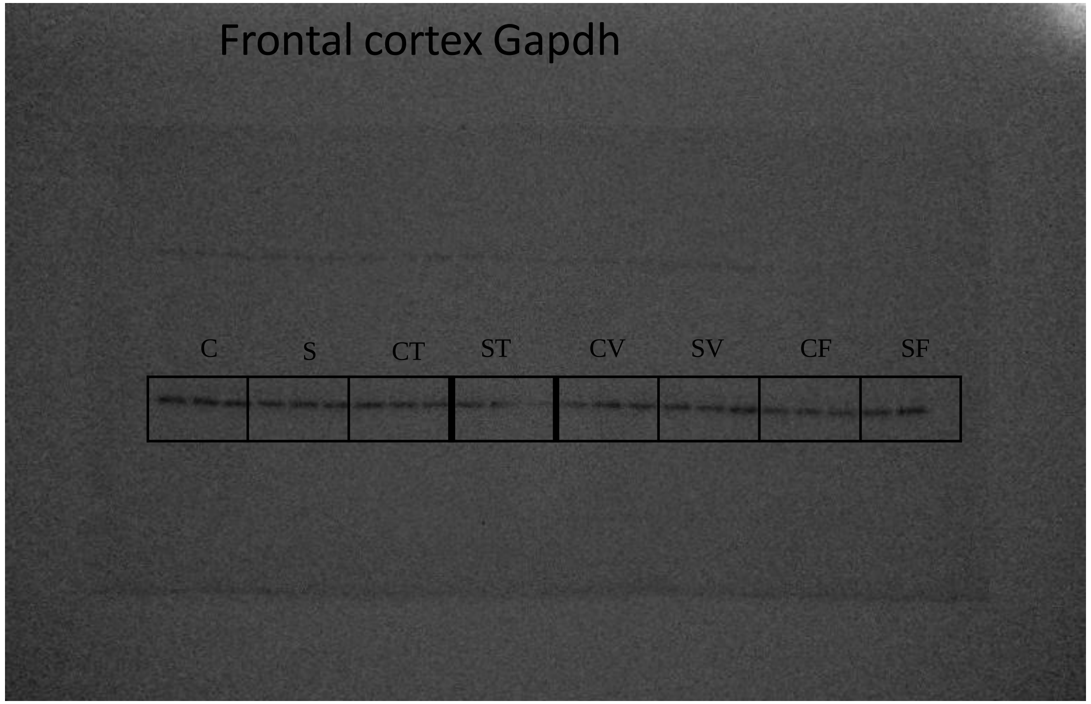

C – CONTROL+VEH
S – STRESS+VEH
CT - CONTROL+TIA
ST – STRESS+TIA
CV – CONTROL+VEN
SV – STRESS+VEN
CF – CONTROL+FLU
SF – STRESS+FLU

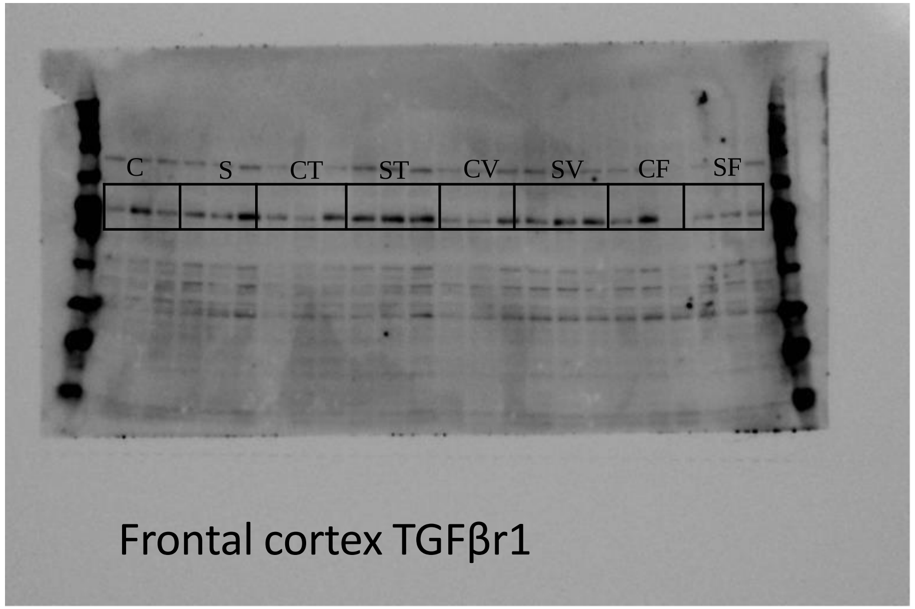

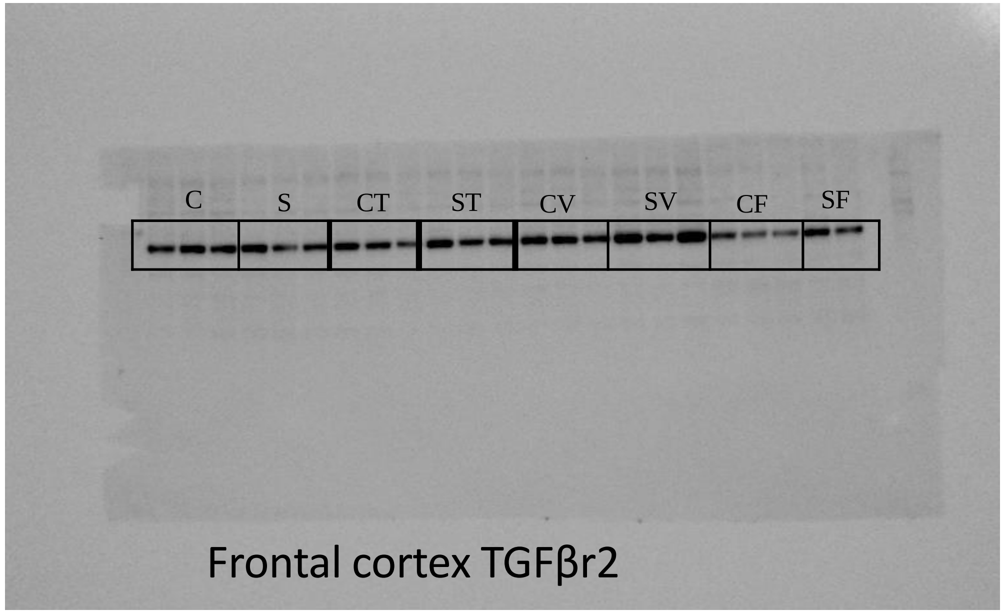

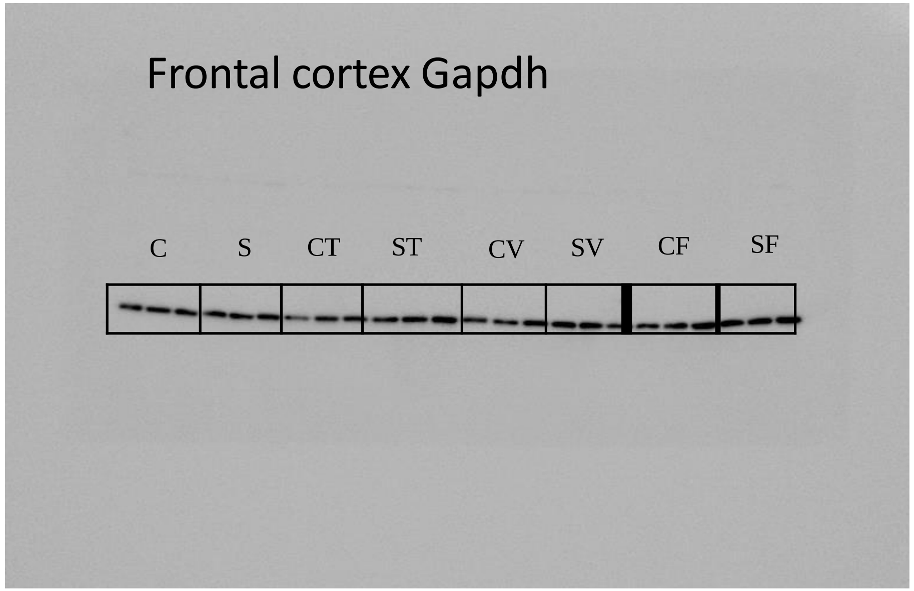

C – CONTROL+VEH
S – STRESS+VEH
CT - CONTROL+TIA
ST – STRESS+TIA
CV – CONTROL+VEN
SV – STRESS+VEN
CF – CONTROL+FLU
SF – STRESS+FLU

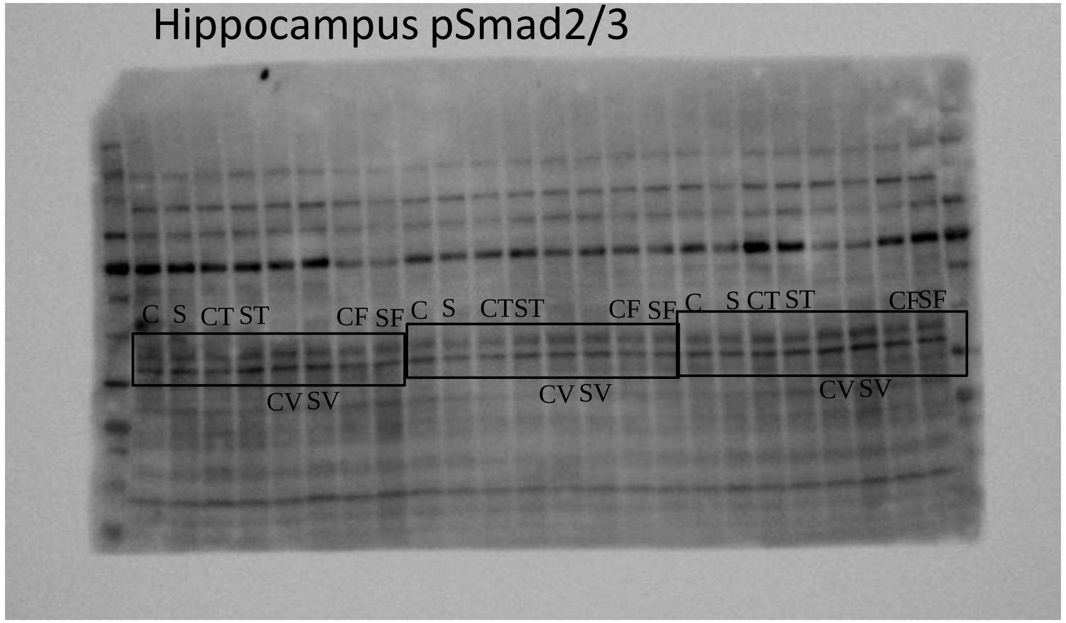

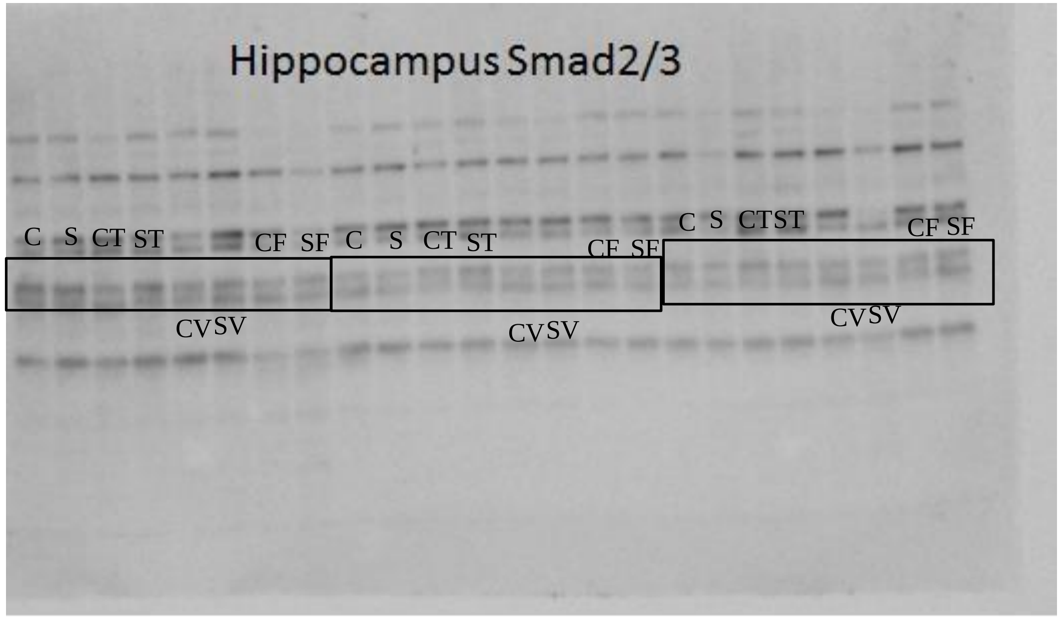

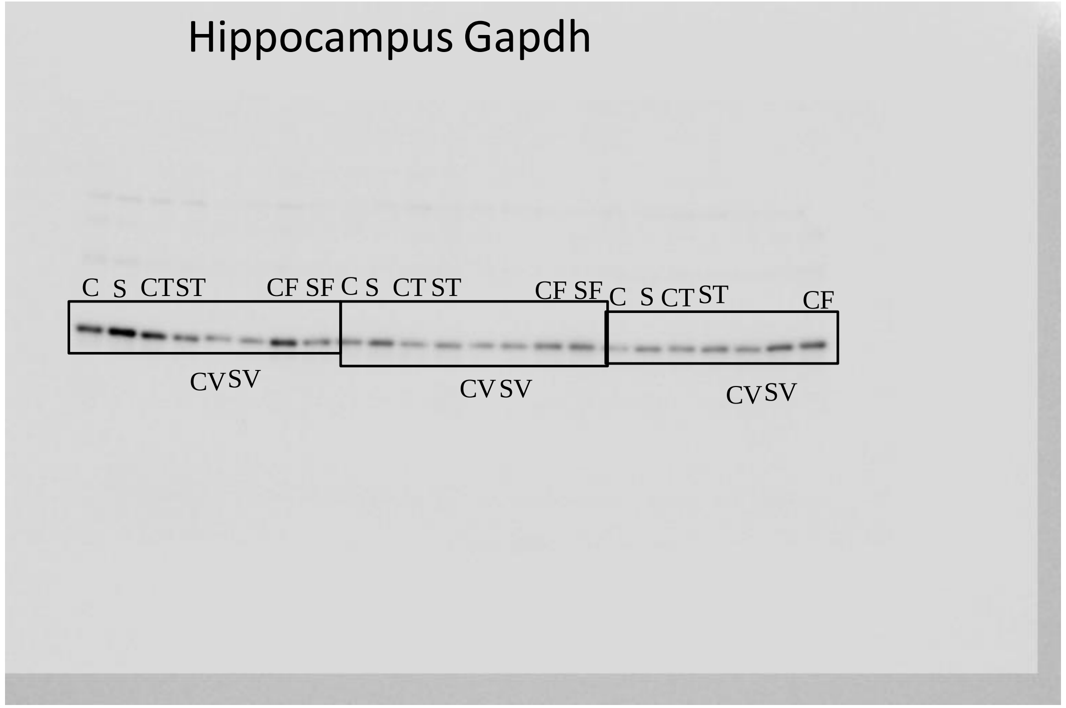

C – CONTROL+VEH
S – STRESS+VEH
CT - CONTROL+TIA
ST – STRESS+TIA
CV – CONTROL+VEN
SV – STRESS+VEN
CF – CONTROL+FLU
SF – STRESS+FLU

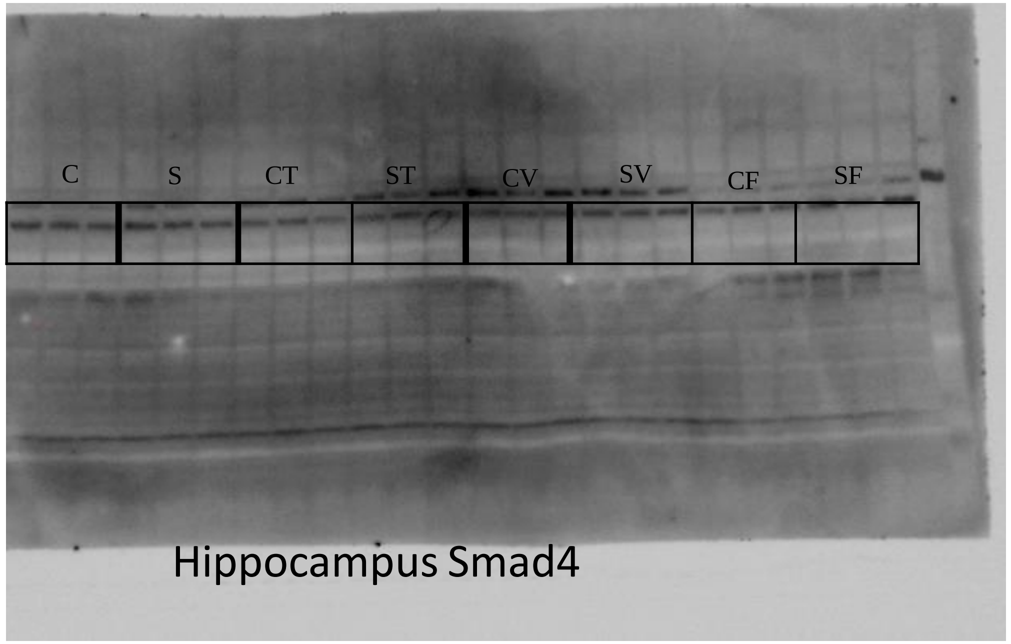

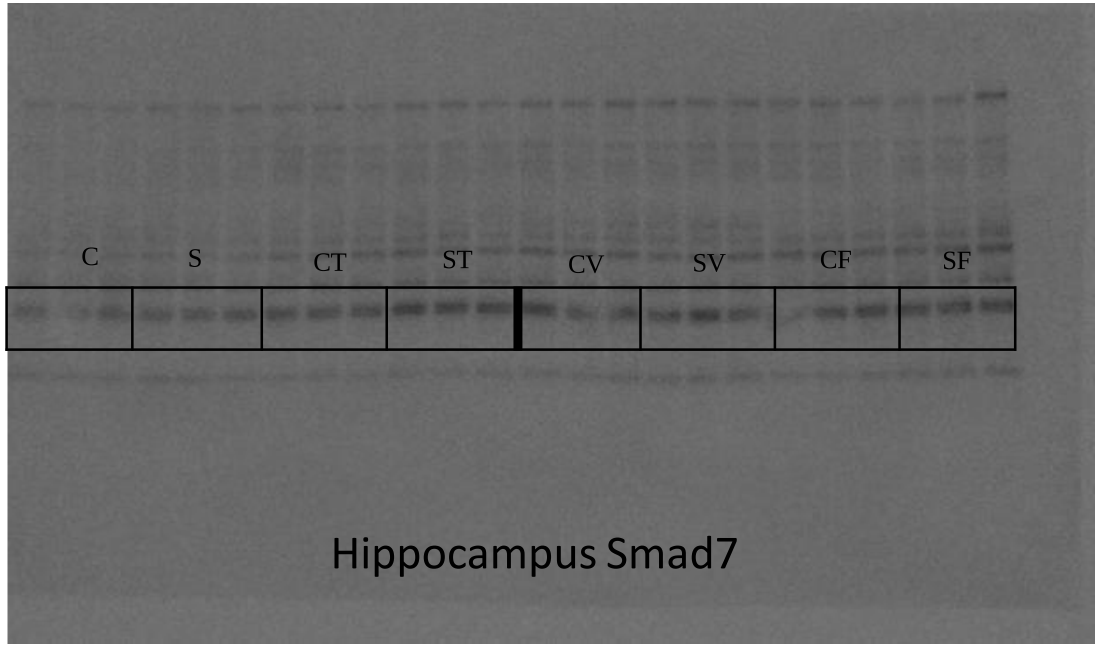

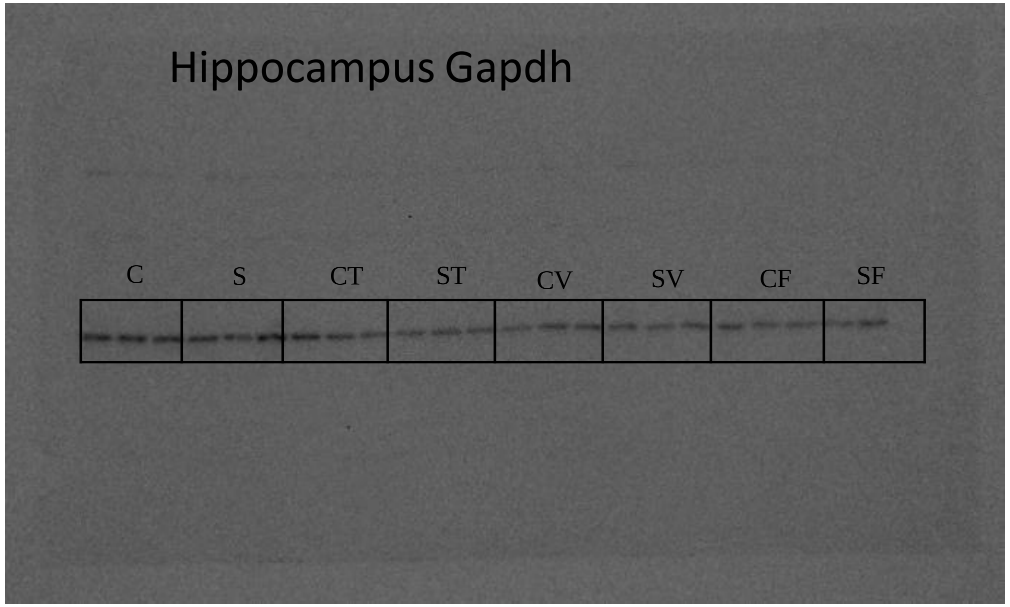

C – CONTROL+VEH
S – STRESS+VEH
CT - CONTROL+TIA
ST – STRESS+TIA
CV – CONTROL+VEN
SV – STRESS+VEN
CF – CONTROL+FLU
SF – STRESS+FLU

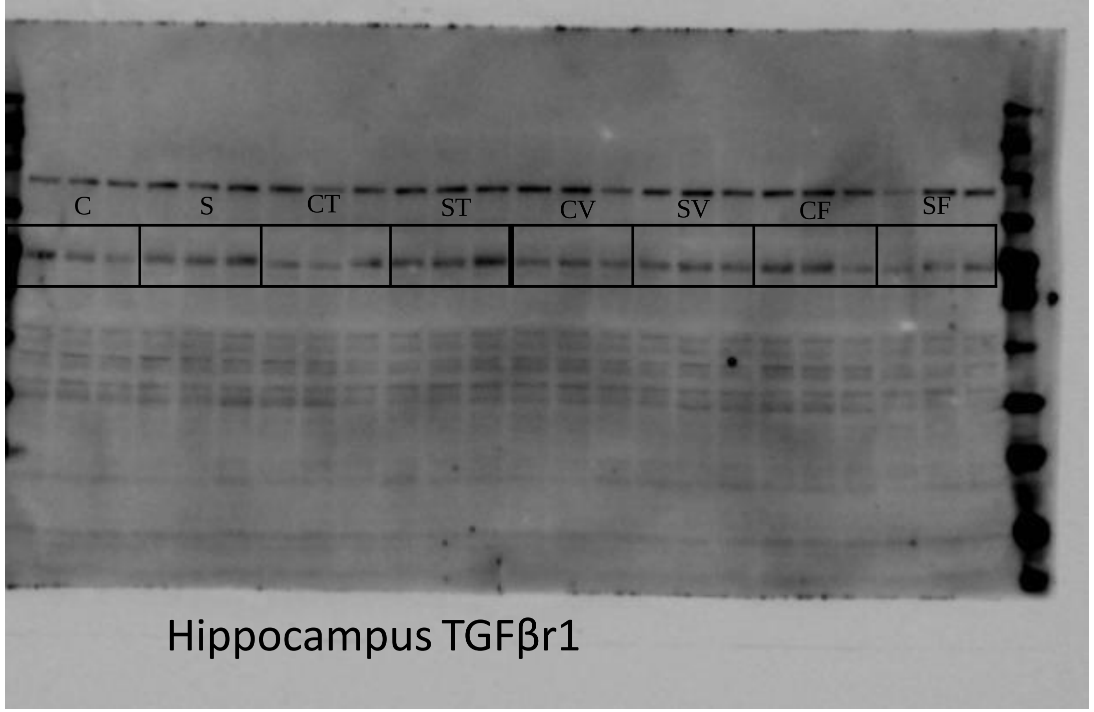

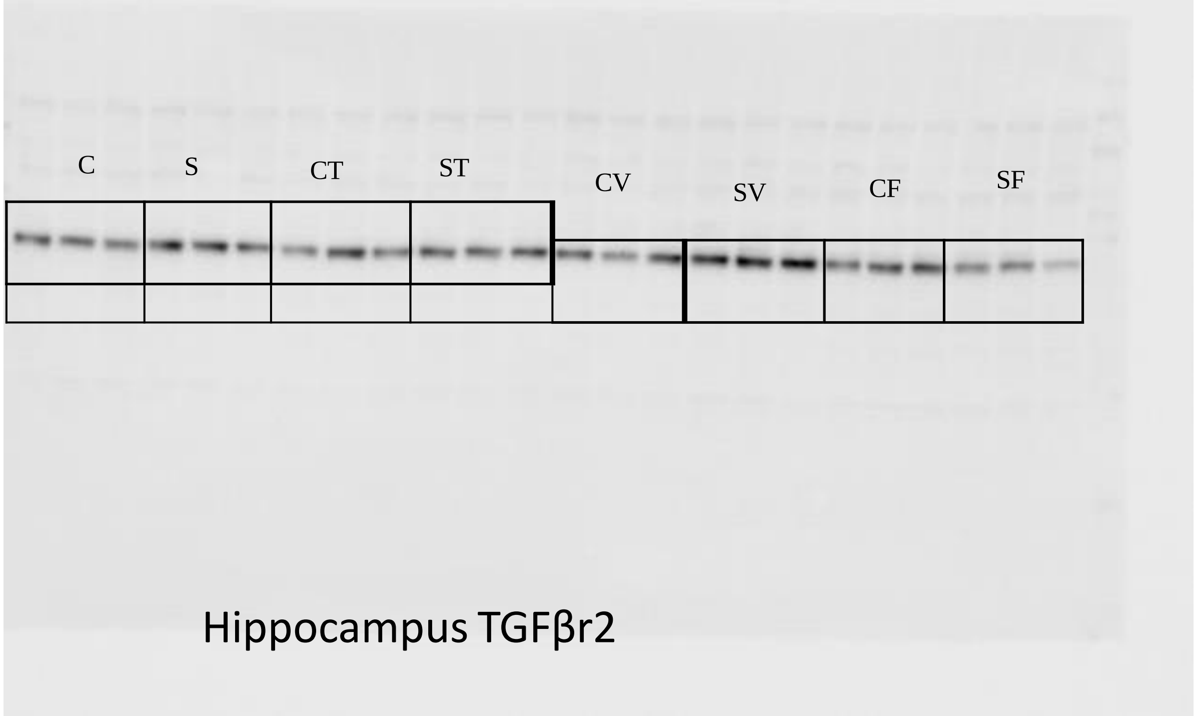

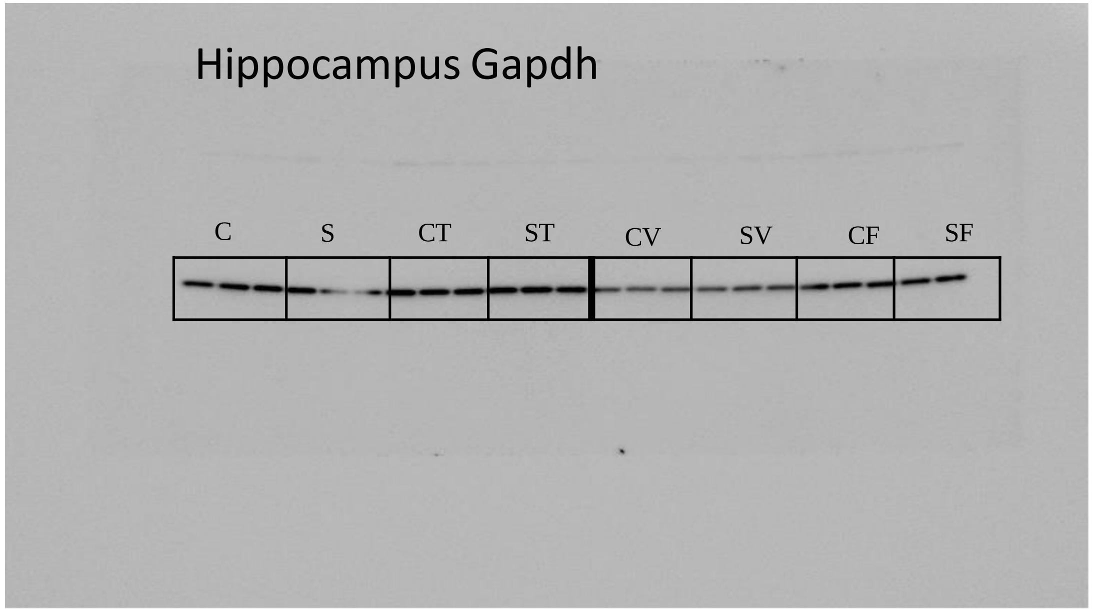

C – CONTROL+VEH
S – STRESS+VEH
CT - CONTROL+TIA
ST – STRESS+TIA
CV – CONTROL+VEN
SV – STRESS+VEN
CF – CONTROL+FLU
SF – STRESS+FLU
